# Flourishing together: research protocol for developing methods to better include disabled people’s knowledge in health policy development

**DOI:** 10.1186/s12913-022-08655-2

**Published:** 2022-10-17

**Authors:** Rachelle A. Martin, Angelo P. Baker, Kirsten Smiler, Lesley Middleton, Jean Hay-Smith, Nicola Kayes, Catherine Grace, Te Ao Marama Apiata, Joanne L. Nunnerley, Anna E. Brown

**Affiliations:** 1 Department of Medicine, Rehabilitation Teaching and Research Unit (RTRU) | Te Whare Whakamātūtū, University of Otago, Wellington South, PO Box 7343, Wellington, 6242 New Zealand; 2 Hā-i-mano | Burwood Academy Trust, Christchurch, New Zealand; 3 Health Services Research Centre, Victoria University of Wellington, Wellington, New Zealand; 4 Centre for Person Centred Research, Auckland University of Technology, Auckland, New Zealand; 5 Whānau Whanake, Christchurch, New Zealand; 6 Department of Orthopaedic Surgery and Musculoskeletal Medicine, University of Otago, Christchurch, New Zealand; 7 Toi Āria | Design for Public Good, Massey University, Wellington, New Zealand

**Keywords:** Disability, Participatory, Co-production, Health policy, Policy planning, Health outcomes, Housing, Realist research

## Abstract

**Background:**

To positively impact the social determinants of health, disabled people need to contribute to policy planning and programme development. However, they report barriers to engaging meaningfully in consultation processes. Additionally, their recommendations may not be articulated in ways that policy planners can readily use. This gap contributes to health outcome inequities. Participatory co-production methods have the potential to improve policy responsiveness. This research will use innovative methods to generate tools for co-producing knowledge in health-related policy areas, empowering disabled people to articulate experience, expertise and insights promoting equitable health policy and programme development within Aotearoa New Zealand. To develop these methods, as an exemplar, we will partner with both tāngata whaikaha Māori and disabled people to co-produce policy recommendations around housing and home (kāinga)—developing a nuanced understanding of the contexts in which disabled people can access and maintain kāinga meeting their needs and aspirations.

**Methods:**

Participatory co-production methods with disabled people, embedded within a realist methodological approach, will develop theories on how best to co-produce and effectively articulate knowledge to address equitable health-related policy and programme development—considering what works for whom under what conditions. Theory-building workshops *(Phase 1)* and qualitative surveys *(Phase 2)* will explore contexts and resources (i.e., at individual, social and environmental levels) supporting them to access and maintain kāinga that best meets their needs and aspirations. In *Phase 3,* a realist review with embedded co-production workshops will synthesise evidence and co-produce knowledge from published literature and non-published reports. Finally, in *Phase 4*, co-produced knowledge from all phases will be synthesised to develop two key research outputs: housing policy recommendations and innovative co-production methods and tools empowering disabled people to create, synthesise and articulate knowledge to planners of health-related policy.

**Discussion:**

This research will develop participatory co-production methods and tools to support future creation, synthesis and articulation of the knowledge and experiences of disabled people, contributing to policies that positively impact their social determinants of health.

## Background

To optimise equitable health outcomes for disabled people,^1^ there is a need to develop participatory partnership approaches supporting the creation, synthesis and articulation of their knowledge and experiences for use by health-related policy planners and programme developers [1–3]. It has been argued that developing knowledge sharing practices that entrust and empower disabled people to articulate their concerns and recommendations will contribute to their voices being more meaningfully included in future health-related policy and programme development within Aotearoa New Zealand (hereafter referred to as NZ) and internationally [1, 4, 5].

In 2013, within NZ, an estimated 24% reported experiencing disability [6], with projections of an increase to 27% by 2038 [7]. Māori have higher age-adjusted rates of disability (32%) than non-Māori (24%) [6]. Strong evidence demonstrates inequity of outcome, unmet need, and difficulty accessing health-optimising systems and services for disabled people [8, 9] and particularly tāngata whaikaha Māori [10–13] within NZ.

It is increasingly recognised that approaches to improving the health and wellbeing of people living with the experience of disability must ensure cross-sector population-level policy and programme development [14–17]. The Life Course Model [18] defines health as ‘an emergent set of capacities that develop over a lifetime to enable individuals to interact successfully with their biological, physiological, psychological, and social environments and realise their potential and wellbeing' (p471). However, within the social model of disability, it is also recognised that disability is created by these same social, cultural and economic structures [19]. Therefore, these systems and structures (e.g., related to income, employment, education, housing, transportation, social cohesion, etc.) act as social determinants of health and wellbeing. A Māori model for health promotion, the Meihana Model [20] locates a whānau (family) and individual within a traversing sea vessel. Within this model, the hauora (wellbeing) of those on board are in relationship to the dynamic environmental forces, which include impacts of colonization and social determinants. However, despite recognising complex interacting factors determining health and disability outcomes, research addressing systemic issues impacting a person’s experience of disability is lacking [14]. There is also a need to develop population-level solutions addressing inequities for disabled people [15, 17].

To positively impact the social determinants of health, people who experience disability need to be included in developing policies and programmes that affect them [9, 15, 21–23]. Co-designed and participatory methods for developing policy and programmes are thought to contribute to more responsive and effective planning and service design, thereby enhancing health, wellbeing, and equity outcomes [21, 24]. Additionally, policies are prone to fail so long as unique aspects of indigenous identity, such as collective health, are not recognized [25]. Co-production methods that strengthen social capital, citizenship and create spaces for dialogue [26, 27] have the potential to improve the quality and responsiveness of health-related policies and programmes [3, 28]. There is also a growing consensus that entrusting and empowering people to co-produce meaning is a critical mechanism for ensuring that research contributes to changes in practice [29–31].

Despite the development of guidelines to support community engagement with people experiencing disability within NZ [32], disabled people continue to feel that they have not been able to contribute to the development of policy and programmes related to social determinants of health impacting them [33–35]. Additionally, policies and programmes related to the social determinants of health are also underdeveloped, with a history of rhetoric that has meant they have struggled to be turned into actions generating sustained attention and political success [36–38]. Policy planners, like researchers [39], also describe challenges when attempting to consult in ways that ensure broad representation of the diverse experiences of disabled people while also gathering and collating their knowledge, expertise, and experiences in a synthesised and useable form. Additionally, there is considerable critique of the levels of participation and partnership allowed within these consultation processes [39–44]. The degree of power-sharing appears to significantly impact eventual outcomes and the authenticity (or not) of the participation and contribution [45–47].

Within NZ, Te Tiriti o Waitangi establishes a partnership between the Crown and Māori—as strategically outlined within the Whakamaua Māori Action Health Plan [48]. The NZ Disability Strategy [49] and Action Plan [50] highlight the need to honour that partnership by developing greater involvement of tāngata whaikaha Māori and disabled people in policy and service development. Recent reforms within NZ’s health system have led to the formation of a Ministry for Disabled People [51]. This Ministry, currently in an establishment phase, is intended to support the national implementation of the Enabling Good Lives approach in alignment with the principles and approaches of Whānau Ora [52]. The NZ government has also indicated that Ministry governance and operationalisation be based on partnership between the disability community and government and will give ‘full effect to the voice of disabled people, families, and whānau’ [53] within the health and disability system. This approach is consistent with the United Nations Convention on the Rights of Persons with Disabilities^2^ [54] and the Declaration on the Rights of Indigenous Peoples [55]. However, despite the recent development of a ‘Disability Toolkit for Policy’ [56], it is currently unclear how these aspirations will be operationalised. This research will contribute to developing methods that can facilitate participatory partnership approaches, enabling disabled people to be meaningfully included in decision-making processes and improving the responsiveness of health-related policies within NZ’s health and disability system transformation.

### Kāinga needs and aspirations as a health-related policy exemplar

One specific health-related issue that dominates the concerns of people experiencing disability is that of kāinga [57]. Kāinga encapsulates aspects of both house and home – including a sense of place and community, where one’s collective identity is lived out. Therefore, this policy exemplar is interested in the ability of tāngata whaikaha Māori and disabled people to access suitable housing and their ability to create a home that meets their needs and aspirations [58, 59]. The focus on kāinga as an exemplar emerged from discussions with disabled people and policy planners who highlighted kāinga as a priority for clear policy direction within NZ. Disabled people frequently experience the compounding effects of limited incomes and low levels of paid employment, making them especially vulnerable to poor housing outcomes [6, 7, 60]. Housing as a social determinant of health is well established [61]. Additionally, the NZ government has conceived their role in providing kāinga, ensuring the ‘quality, accessibility, size, and features of our homes support people and families to live healthy, successful lives’ [62].

However, despite clear evidence linking the physical attributes of housing to health [61, 63], the mechanisms by which housing contributes to health outcomes are complex [64]. There is growing theoretical and empirical evidence linking the less tangible aspects of housing (‘the psychosocial benefits of home’) to wellbeing [64, 65]. Therefore, there is a need to consider attributes of houses beyond being physically accessible, warm, safe and affordable, to also include people’s accessibility to homes within communities that allow autonomy, the development of self-identity, socialisation, and status [65, 66].

#### Study objectives

In this research, we are partnering with tāngata whaikaha Māori and disabled people to achieve two key objectives:

1. In partnership with disabled people, we will develop innovative methods and tools empowering people experiencing disability to co-produce and effectively articulate knowledge to inform equitable health-related policy and programme development within NZ.
2. As an exemplar, we will synthesise evidence and co-produce knowledge to inform the development of equitable health-related housing and home policies within NZ. We will do this by developing a nuanced understanding of how contexts and resources (i.e., at individual, social and environmental system levels) interact to support tāngata whaikaha Māori and disabled people to access and maintain kāinga that best meet their needs and aspirations.

## Methods

### Overview of research approach

This study uses participatory co-production methods [3, 67, 68] within a realist methodological approach [69]. Realist methods unpack the ‘black box’ of complex programmes and policies by developing theoretically-based understandings of what works for who, in which contexts, to what extent, and how [69]. Co-production methods [3, 67, 68] will collaboratively generate knowledge in partnership with people with lived experience, opening dialogue for new ways of thinking and potentially challenging dominant discourses within policy planning praxis. Realist methods are advocated to better support policy development [70], and their use alongside participatory approaches is, for example, demonstrated by Langley and colleagues [3, 68]. The University of Otago Human Ethics Committee (Health) has provided ethical approval for this research [H21/099].

Table 1 presents the results of a desktop exercise considering the range of theories and approaches underpinning participatory co-production methods within the context of disability and housing policy. The purpose was to identify potential explanations that might be observed and refined further by the research. Constructing this table follows the programme theory building stage characterising the start of any realist research approach [71]. We distinguished between macro-, meso- and micro-system levels and explanations for: (1) how equitable health-related housing and home policies are expected to emerge from the inclusion of the diverse experiences of disabled people; (2) how co-production works; and (3) the value of different contexts with respect to housing and home for different individuals. These initial programme theories will be used to examine and synthesise the diverse evidence collected in the research phases outlined below.

**Table 1:** Initial theoretical concepts informing the research

System levels	Concepts underlying initial programme theories
***Macro-system*** How are equitable health-related housing and home policies expected to emerge from the inclusion of the diverse experiences of disabled people?	Policy studies accounts related to international expectations and evidence of inequity for housing policy change in NZ—shifting from market-led paradigms to enriched relationships between private, public and community. Explanations stressing the importance of health's social and economic determinants to achieve cross-sector population-level policy and programme development. Includes the potential for needs to change across the life course and broader understandings of the social model of disability. Dynamics for tāngata whaikaha Māori sitting within desires for Māori control and authority as a solution to confronting persistent inequities and honouring Te Tiriti o Waitangi partnership—with initial steps made towards a National Māori Housing strategy developed in partnership with Māori.
***Meso-system*** How is co-production expected to work to improve outcomes?	Theories that co-production methods strengthen social capital, citizenship and create spaces for dialogue. Realist-based and implementation research on the challenges involved in participatory research. Broader insights linked to social innovation, user-centred design and NZ experiences of co-design. Theories of how a research team of Māori and non-Māori can best operate acknowledging different knowledge systems, each with their own internal logics (e.g., negotiated spaces model).
***Micro-system*** Recognition of the diversity of individuals involved, and different sense-making with respect to housing and home	Theories related to the diversity and intersectionality of experiences, e.g., Māori, disability and other social experiences relevant to housing and home, such as gender, family, socioeconomics, community and rurality. Growing theoretical and empirical evidence linking the less tangible aspects of housing (‘the psychosocial benefits of home’) to wellbeing. Dynamics for tāngata whaikaha Māori: Kaupapa Māori research on how home is perceived. Frameworks that distinguish between choice, voice and representation as options to support individuals in identifying solutions.

### Overview of research phases

**Fig. 1:**
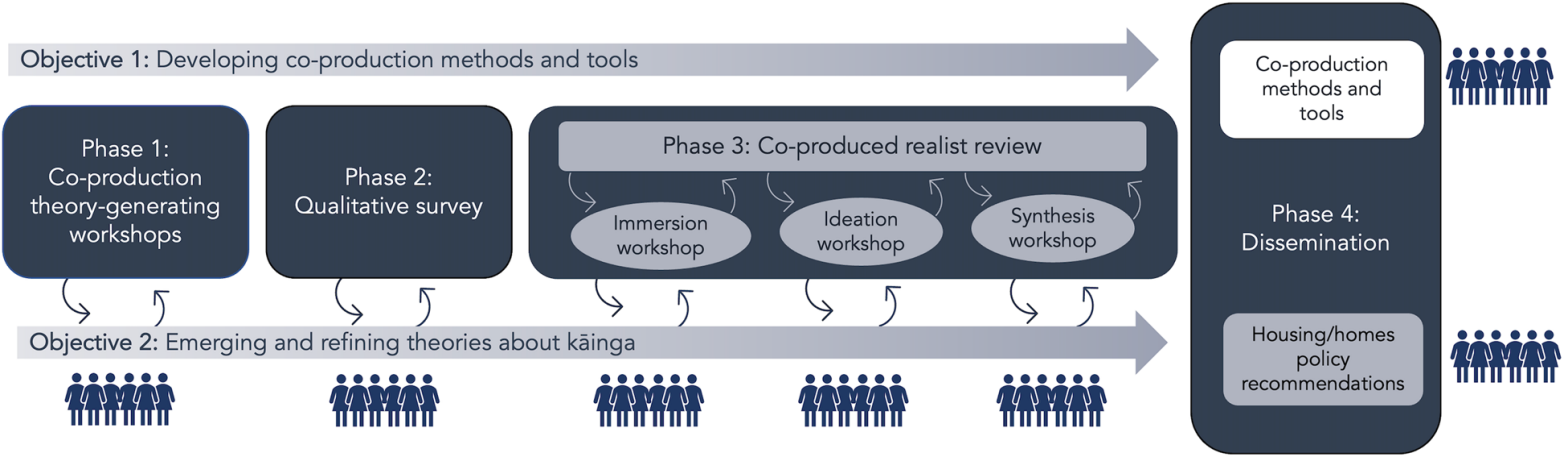
Overview of research design

The four-phase research programme (Fig. 1) will be conducted within NZ. In *Phase 1*, co-design theory-building workshops with tāngata whaikaha Māori and disabled people (*n* = 20) will postulate theories about how housing works (or not), for whom, and in what circumstances to improve health-related outcomes. In *Phase 2*, qualitative survey responses (*n* = 200) gathered from disabled people across NZ will explore contexts and resources (i.e., individual, social and environmental) that support them to access and maintain kāinga, meeting their needs and aspirations. In *Phase 3,* a realist review with embedded co-production workshops with tāngata whaikaha Māori and disabled people will synthesise evidence and generate knowledge to inform the development of equitable housing policy. Finally, in *Phase 4*, data from all phases will be synthesised to develop two key research outputs for dissemination: (1) housing policy recommendations and (2) the articulation of innovative co-production methods and tools that empower disabled people to create, synthesise and articulate knowledge to planners in other policy areas within NZ.

#### Fulfilment of Objective 1 across the research

To capture how co-production activities work in Phases 1–3, workshop facilitators will keep field notes, and collect audio, video and photographic data. At Phase 3 completion, focus groups will explore how various methods are experienced by disabled people across different research phases. To support method development, we will work with designers across the project, including a design student undertaking a Master's thesis who will specifically develop embedded co-production methods and activities within the realist review (Phase 3). We will also fund (a) a Māori graduate or postgraduate student to review Māori models used within disabled advisory spaces and (b) a student with the lived experience of disability to review international disability engagement models (including with indigenous populations). In Phase 4, these models and focus group data will be synthesised to inform future co-production and engagement practices. The participatory approaches, co-production tools, and resources developed and used across the project will be collated and disseminated to inform co-production processes between disabled people and policy planners in other policy areas.

### Inclusion and exclusion criteria of disabled people across research

We will include tāngata whaikaha Māori and disabled people (and their support person or whānau) aged over 18 years of age, with at least one impairment of body structure or function that results in an experience of disability (self-identified) who can communicate (in person or online) with or without the support of a communication device or support person. We are not including disabled people with a mental health condition as the primary health condition contributing to their experience of disability.

### Positioning concerning defining disability

There is debate regarding what disability is and, depending on the definition, who qualifies as experiencing disability [72, 73]. People experiencing disability and disability advocates argue strongly that disability is not a ‘problem’ located within a person's body. Instead, it occurs when personal and environmental factors restrict the opportunities of people with health impairments or conditions [74]. We have kept our definition broad (i.e. people experiencing disability) and prioritised the ability of any person with health-related impairments to self-identify as experiencing disability. Historically, research limiting participation by health conditions has resulted in the ‘usual suspects’ taking part, often excluding people with less common conditions [46]. Self-identification ensures we do not endorse the non-disabled discourses of what is (or is not) the voice of disabled. However, we acknowledge that, in keeping the inclusion criteria broad, the needs and aspirations of disabled people within this research will be varied. Nevertheless, realist research approaches allow us to explicitly unpack nuance and diversity of experience.

### Positioning concerning accessibility requirements

Accessibility is a key consideration across all phases, with careful consideration given to how methods are adapted to meet a range of participants' accessibility needs. As part of Phase 1 and in qualitative survey development in Phase 2, we will explore ways inclusion can be achieved for diverse participants, developing resources and tools to facilitate full participation. Throughout, we will offer alternative formats for accessing information (e.g. easy read, large print, audio) and responding (e.g. verbal, written, online, in-person, proxy) in line with best practice [75] to support participation and the full and nuanced expression of people’s experiences. For Phases 1 and 3, we have opted for nationwide recruitment for the disabled person co-production team to ensure broader representation, especially around kāinga experiences. The qualitative survey (Phase 2) also provides accessibility and participatory advantages affording greater control, less burden and more flexibility for participants [76].

### Positioning concerning tāngata whaikaha Māori

The project is conceptualised to improve health outcomes for Māori explicitly – directly in relation to housing policies and longer term, by ensuring tāngata whaikaha Māori can fully partner and participate in the planning of other health-related policies. We are responding to demonstrated Māori health outcome inequity compared to non-Māori, with compounding inequities for those who experience disability [25]. Concerning kāinga, the legacy of ongoing colonisation forcing urbanisation and the loss of land [77] means that tāngata whaikaha Māori are disproportionately vulnerable to housing issues. This is further compounded for disabled Māori, whose housing vulnerability is more significant than for either non-disabled or non-Māori [25, 78]. This research integrates the principles of Te Ara Tika [79] and Kaupapa Māori [80] within the design. These frameworks provide guiding principles for engaging in respectful research with Māori, including ensuring that engagement, relationship building and shared power in each stage of the research process occurs—from conceptualisation through to dissemination of findings—to ensure the research is culturally safe, relevant, and of benefit, to Māori communities [79]. We have engaged in whakawhanaungatanga over two years, in discussion with Māori agencies and tāngata whaikaha, to co-design the research aims and methods. The Māori researchers have extensive experience working in community, academic and social enterprise settings – focused on supporting health and wellbeing. As such, they retain strong relationships with a wide network of Māori leaders and organisations and will support the translation and utilisation of findings within Māori health, disability and advocacy contexts.

### Project governance

**Fig. 2:**
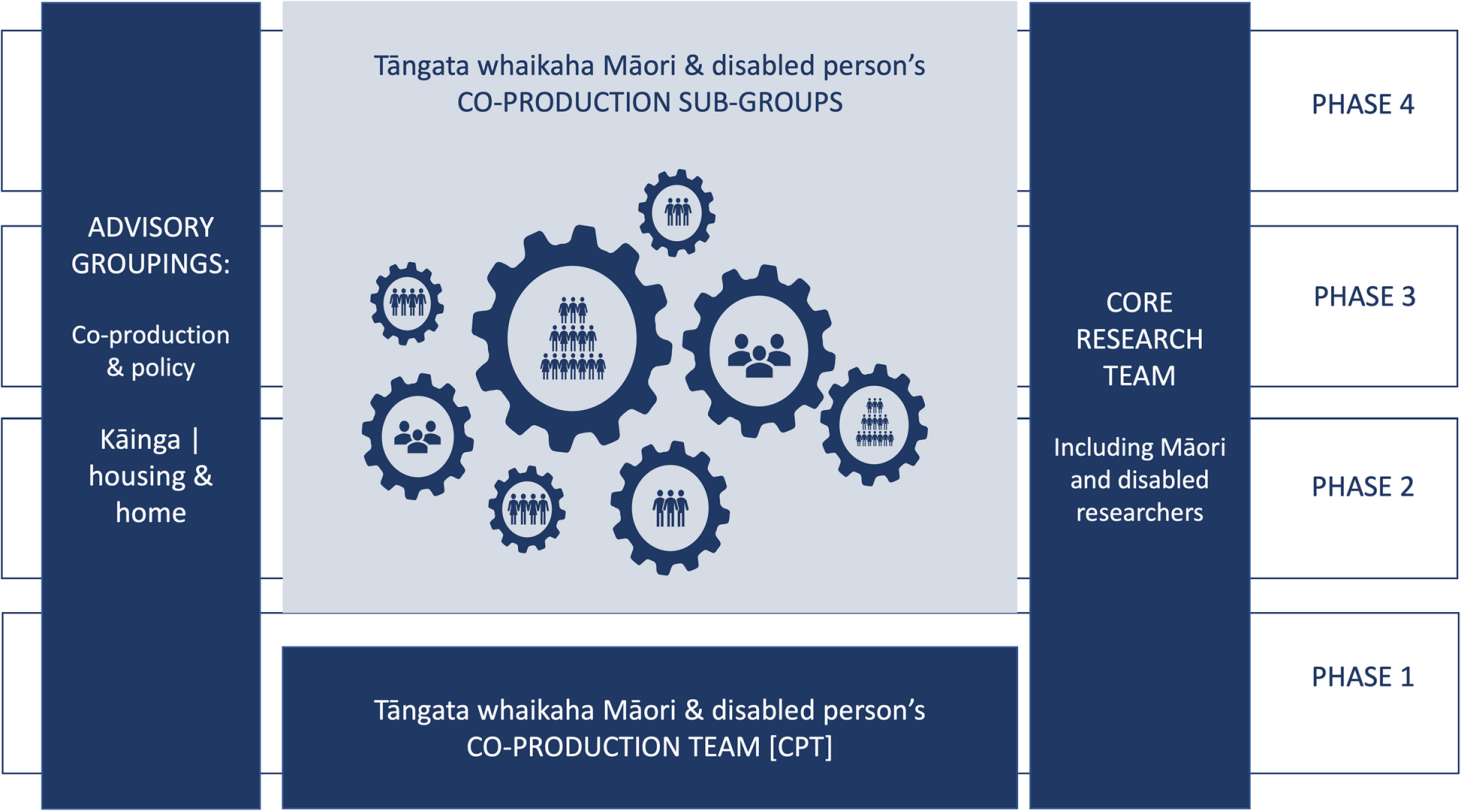
Overview of project governance and co-production research teams

As overviewed in Fig. 2, three groupings will contribute to the creation, synthesis, articulation, and dissemination of new knowledge across all phases of the research project: (1) two advisory groupings, (2) tāngata whaikaha Māori and disabled person’s co-production team, and (3) the core research team. The core research team is intentionally diverse in experience and knowledge, including Māori [CG, TAMA, KS]; people who experience disability [CG and AB]; co-production and co-design [RM, JN, NK, AB]; realist [RM, LM, KS, NK, JHS]; and policy research expertise [LM, KS]. Across the project's life, the two advisory groupings related to (1) co-production and policy and (2) kāinga and disability, will provide accountability and ethical oversight and offer advice regarding research applicability and relevance to NZ health, disability and social service contexts.

#### Disabled person’s co-production team (CPT)

In Phase 1, the CPT will consist of 20 members. The intention is to continue to build the CPT membership so that in later phases, the original CPT members will be supported to develop sub-groups, each focused on a unique aspect of kāinga. For example, original CPT members may co-lead groups focused on the needs of disabled people moving away from their parent's homes, living rurally, experiencing predominantly physical access needs, requiring specific communication needs to be met, tāngata whaikaha Māori, or those who are aging.

The CPT members will have two roles within the project. First, they will be *lived experience experts* who contribute to developing policy recommendations related to kāinga, determining the focus and scope of the kāinga research project (in fulfilment of Objective 2). Second, they will be *research participants* who voluntarily contribute their perspectives about the co-production processes, strategies and tools developed and used within the project (in fulfilment of Objective 1).

#### CPT recruitment

We will recruit CPT members to ensure ethnic, age and citizenship representation and diversity. Recruitment will begin through disability advocacy organisations and groups already collaborating with the project. Care will also be taken to ensure that tāngata whaikaha Māori and those not typically involved in advocacy/advisory roles are informed of the opportunity to participate (e.g., people with communication difficulties, disabled youth, and those with ‘invisible disability’).

We have opted for nationwide recruitment for the CPT rather than one locally-based team with face-to-face meetings. We have reasoned that the processes and tools developed working alongside a dispersed CPT will have more future applicability for central and local government, agencies and organisations. Online (synchronous) and offline (asynchronous) strategies will also allow for broader representation around kāinga issues due to location (e.g., more accessible for those living rurally or who experience difficulties with transportation), increased opportunities for Māori recruitment, and future-proofing against possible disruption due to a pandemic or other environmental conditions. However, we realise that this approach may continue to exclude key groups in disability research, and that some methods of technology-based co-production may be more challenging for those with specific communication requirements. We will continue developing our awareness of and responsiveness to proactively addressing the concerns inherent in a dispersed CPT.

People will be asked to express their interest in whatever format best suits them (e.g., written, audio, video, messaging application). We will then contact each person expressing interest to establish their expertise, context, and accessibility needs. Recruitment to the CPT will be a values-driven choice to balance voices – ensuring the inclusion of tāngata whaikaha Māori and diversity of current and past housing experiences.

### Phases of research

#### Phase 1: co-production theory-generating workshops [3, 81]

Co-production workshops with the CPT will generate hypotheses about how housing works (or not), for whom, and in what circumstances to improve health-related outcomes. The generated initial programme theories will postulate how individual, social and environmental resources support (or not) disabled people to access and maintain kāinga that best meet their needs and aspirations. Interactive knowledge generation methods will be developed as part of this research, based on the methodological work of Langley et al. [67]. Before workshop 1, CPT members will be given the opportunity to interact asynchronously with participatory and creative materials sent in advance. Synchronous discussions as part of two 2-h online workshops will then co-produce further data to inform the initial programme theories being refined across the research and determine the research’s focus concerning kāinga. In workshop 2, the CPT will use synthesised Phase 1 data to develop the qualitative survey (Phase 2) and realist review search strategy (Phase 3).

#### Phase 2: qualitative survey [76]

In Phase 2, qualitative survey responses (*n* = 200) gathered from disabled people across NZ will further produce rich and complex accounts about the contexts and resources (i.e., individual, social and environmental) that support them to access and maintain kāinga that meet their needs and aspirations more generally. A sample size of 200 (at least 20% tāngata whaikaha Māori) is based on feasibly achieving (i.e., funds, time) a large and diverse sample allowing us to conceptualise and assess richness in the whole dataset. It is anticipated that the qualitative survey can provide an accessible and resource-efficient means of gathering nuanced, in-depth experiences and meanings sufficient for qualitative analysis – ensuring that diverse voices, including those who are often excluded from more typical stakeholder engagement fora, can participate.

##### Recruitment

via NZ disability organisations, health or support providers, social housing providers (including within Māori communities), and care agencies. Social media advertisements and snowball sampling will also be used. Purposive recruitment will occur as needed to ensure diverse representation (e.g., ethnicity, range of impairments, urban versus rural) rather than to achieve saturation [76].

##### Data collection

Survey design will be based on Phase 1 findings. Data will be generated via open-ended, text-based questions and collected in ways responsive to participants' communication preferences and capabilities—including online self-filled, postal, or in-person, with a research assistant typing responses verbatim. As part of the survey design, we aim to develop qualitative survey methods that promote inclusivity, accessibility and responsiveness to tikanga Māori. While question phrasing and content are not yet known, we anticipate the survey will comprise approximately ten open-ended questions. Quantitative demographic data will also be collected (e.g., age, ethnicity, health condition and impairments, current housing status), along with the Washington Group Short Set disability questions [82].

##### Analysis and output

Demographics will be summarised descriptively. The core research team will undertake qualitative analysis informed by realist logic (i.e., coding for context, mechanism and outcome configurations). Data analysis and interpretation will also be supported by CPT engagement with asynchronous materials and in the second online workshop. Findings from Phase 2 will further refine the initial programme theory initially developed in Phase 1.

#### Phase 3: co-produced realist review [70, 83]

In Phase 3, realist review methods [70, 83] with embedded co-production workshops [3, 67, 68] will review and synthesise white literature (i.e., peer-reviewed, published research) and grey literature (e.g., Waitangi Tribunal reports, NZ government strategy documents and reports). Table 1 outlines possible theoretical underpinnings of health-related housing policy development, and elements of the theories provide a platform for the steps listed below.

##### Realist review methods

A realist synthesis [83] of existing empirical and theoretical literature will be conducted using the following steps:

1. *Scoping phase:* Based on Phase 1 and 2 findings, a comprehensive search strategy will be developed in conjunction with an information search specialist.
2. *Searching phase:* Iterative cycles of engagement with literature based on the initial search strategy using a range of databases and grey literature searching strategies. Additional searching will ‘fill the gaps’, exploring new theoretical areas for development.
3. *Document selection:* development of evidence decision matrix based on relevance and rigour.
4. *Data extraction and analysis:* Data will be extracted into evidence tables describing data characteristics. NVivo will be used for realist logic analysis coding. Data analysis within a realist synthesis aims to find evidence of relationships between mechanisms, contexts and outcomes within the data—in this case, to find evidence that supports, refutes and refines the programme theory developed from Phase 1 and 2 findings. Researchers will code data from peer-reviewed documents and grey literature using a shared NVivo file (thus inductively developing a shared coding framework). They will also organise the extracted information into tables, looking for recurring patterns in the contexts, mechanisms and outcomes, and then presenting and exploring tentative findings within the three CPT workshops (thus ensuring the co-construction of shared meaning). We will iteratively refine the initial programme theory by generating and prioritising theoretical concepts based on the literature review.
5. *Development of programme theory* (based on literature) and synthesis with programme theory developed and refined in Phases 1 and 2.

##### Co-production methods

The CPT members will co-produce meaning and contribute to the final analytic product via three co-production workshops.

- *Workshop 1 (Immersion)* will enable the team to be immersed in the literature found in the searching phase.
- *Workshop 2 (Ideation)* will occur in the initial data analysis stage and use creative activities to generate and prioritise concepts.
- *Workshop 3 (Synthesis)* will contribute to synthesising concepts into a developed programme theory.

To facilitate CPT engagement with the data and sense-making, innovative purpose-built tools will be developed as part of this project. A refined programme theory will then be used to develop kāinga recommendations in Phase 4 [84].

#### Phase 4: dissemination and pathways to impact

To disseminate findings, we will develop resources and tools that empower disabled people to create, synthesise and articulate knowledge to planners of health-related policy more generally (Objective 1). In addition, we will collate findings from Phases 1–3 to write a briefing paper outlining housing policy recommendations (Objective 2). Research outputs will focus on optimising the utilisation of findings. They will be disseminated to various stakeholders, including housing policy planners at local and central government departments, disability advocacy organisations, and Māori organisations.

## Discussion

The involvement of disabled people in developing health policy is increasingly being advocated [1, 5, 27]. However, challenges with operationalising this involvement frequently emerge [40–42]. For example, who should be involved, what does involvement look like, and how genuine is the power-sharing? This research seeks to address two specific but interrelated objectives. By addressing these objectives, we want to empower disabled people to participate in policy development within NZ in the future, thereby ensuring their diverse and nuanced needs and aspirations can be more effectively addressed and specifically, in this case, related to housing and home. By addressing the needs and aspirations of tāngata whaikaha Māori and disabled people regarding kāinga, policies can realise improvements in the social determinants of health and health and wellbeing outcomes, and increase the ability of disabled people to participate in and contribute to the community.

Regarding the first research objective, the development of co-production methods within this project aims to address barriers to active and meaningful participation of disabled people in policy development. First are barriers related to consultation process accessibility due to, for example, communication access needs, contribution style preferences, living location, transportation requirements, and technological and digital literacy and accessibility. Second are barriers related to how policy questions are framed within policy planning praxis and the need to challenge dominant discourses, including *what* is being consulted on and *how* consultation occurs. Finally, are issues related to the challenge of providing policy recommendations in synthesised yet still nuanced formats, thereby allowing policy developers to access the knowledge and experience of disabled people more readily. It is anticipated that more inclusive and effective tools can promote the power, prerogative and participation of tāngata whaikaha Māori and disabled people in policy advocacy and consultation, increasing policy relevance and effectiveness. As this research is being conducted during a particular policy window, when significant policy change is more likely given the stronger directions for consultation and co-production emerging from the current NZ health and disability system transformation, it is hoped findings can contribute to more disability-responsive health-related policies within NZ.

Pathways to research impact are evident in the design and conduct of the research itself. Tāngata whaikaha Māori and disabled people have been involved in the conception and design of this project and will determine the final scope of the research in Phase 1. They will co-produce analytical content through all phases of research, will co-design dissemination resources and assist with knowledge translation activities. It is also anticipated that communities of practice could form around ways of engaging and advocating within the CPT members, meaning the disabled people could translate the co-production and knowledge generation learnings to other advocacy spaces they are involved with currently or in the future.

## Data Availability

Data sharing is not applicable to this article as no datasets have been generated or analysed at this stage.

## Abbreviations

BAT: Burwood Academy Trust
CPT: Co-production Team
UNCRDP: United Nations Convention on the Rights of Disabled Peoples
UNDRIP: United Nations Declaration on the Rights of Indigenous Peoples
NZ: Aotearoa New Zealand

## Footnotes

1 *We use ‘disabled people’, ‘tāngata whaikaha’ (a Māori term literally translated as ‘people in the pursuit of empowerment’), or ‘people who experience disability’ in line with affirmative language used by NZ disabled-led organisations and strategy documents.*^*25,26*^* This terminology emphasises the capacities and strengths of the individuals and the ways that society can disable and disempower individuals with impairments.*

2 This research aligns and intersects with the UNCRDP in several ways – but particularly with Article 29 (effective and full participation in the conduct of public affairs) and Article 19 (living independently and experiencing choice in their place of residence) – and will support the Crown’s progress towards realising its responsibilities.

## Glossary

*Aotearoa*: A Māori name for New Zealand.
*Hauora*: A Māori holistic conceptualisation of health or wellbeing which encompasses physical, mental/emotional, social and spiritual wellbeing.
*Kāinga*: A Māori term encapsulating aspects of house and home – including a sense of place and community, where one’s collective identity is lived out.
*Kaupapa*: A Māori term alluding to a topic, matter for discussion, agenda, or programme of work.
*Māori*: The indigenous people of Aotearoa, New Zealand.
*Te Reo Māori*: The indigenous language of Aotearoa, New Zealand.
*Te Tiriti o Waitangi*: Aotearoa New Zealand’s founding document, a partnership agreement between Māori and the British Crown, signed in 1840.
*Tāngata whaikaha*: A Māori term for ‘disabled people’; literally translated as ‘people in the pursuit of empowerment’.
*Tikanga*: Māori customary values, customs, procedures and practices.
*Whakawhanaungatanga*: The process of establishing connection, building relationship, and relating well to others.
*Whānau*: Family; can include extended family and friends who may not have kinship relationships but are like family.
